# Empirical and Theoretical Characterization of the Diffusion Process of Different Gadolinium-Based Nanoparticles within the Brain Tissue after Ultrasound-Induced Permeabilization of the Blood-Brain Barrier

**DOI:** 10.1155/2019/6341545

**Published:** 2019-12-01

**Authors:** Allegra Conti, Rémi Magnin, Matthieu Gerstenmayer, Nicolas Tsapis, Erik Dumont, Olivier Tillement, François Lux, Denis Le Bihan, Sébastien Mériaux, Stefania Della Penna, Benoit Larrat

**Affiliations:** ^1^NeuroSpin, Institut des Sciences de La Vie Frédéric Joliot, Direction de La Recherche Fondamentale, Commissariat à L'Energie Atomique et Aux Energies Alternatives, Université Paris Saclay, Gif-sur-Yvette, France; ^2^Department of Neuroscience, Imaging and Clinical Sciences, Institute for Advanced Biomedical Techniques, G. D'Annunzio University, Chieti, Italy; ^3^Image Guided Therapy, Pessac, France; ^4^Institut Galien Paris-Sud, CNRS, Univ. Paris-Sud, Université Paris-Saclay, Châtenay-Malabry, France; ^5^University Lyon 1, Lyon, France; ^6^Consiglio Nazionale Delle Ricerche, Institute SPIN, UOS L'Aquila, Site CNR-SPIN C/o Università di Chieti-Pescara “G. D'Annunzio”, Chieti, Italy

## Abstract

Low-intensity focused ultrasound (FUS), combined with microbubbles, is able to locally, and noninvasively, open the blood-brain barrier (BBB), allowing nanoparticles to enter the brain. We present here a study on the diffusion process of gadolinium-based MRI contrast agents within the brain extracellular space after ultrasound-induced BBB permeabilization. Three compounds were tested (MultiHance, Gadovist, and Dotarem). We characterized their diffusion through *in vivo* experimental tests supported by theoretical models. Specifically, by estimation of the free diffusion coefficients from *in vitro* studies and of apparent diffusion coefficients from *in vivo* experiments, we have assessed tortuosity in the right striatum of 9 Sprague Dawley rats through a model correctly describing both vascular permeability as a function of time and diffusion processes occurring in the brain tissue. This model takes into account acoustic pressure, particle size, blood pharmacokinetics, and diffusion rates. Our model is able to fully predict the result of a FUS-induced BBB opening experiment at long space and time scales. Recovered values of tortuosity are in agreement with the literature and demonstrate that our improved model allows us to assess that the chosen permeabilization protocol preserves the integrity of the brain tissue.

## 1. Introduction

The *in vivo* characterization of gadolinium-(Gd-) based MRI contrast agent (MR-CA) diffusion within the brain tissue is of great interest for the understanding of drug transport mechanisms in the brain parenchyma, in the framework of the recent pharmaceutical developments targeting entral nervous system (CNS) diseases. Despite increasing efforts and encouraging results, drug delivery to the CNS remains a challenging task. Indeed, the blood-brain barrier (BBB) not only prevents neurotoxic substances from entering the brain but also limits the passage of therapeutic products to the CNS [1, 2]. Many strategies have been studied to overcome this obstacle, including direct injections [3, 4], transient BBB disruption using chemical agents [5, 6], or molecular engineering [7]. More recently, a promising technique has been proposed, allowing the delivery of various compounds to the brain using low-intensity focused ultrasound combined with circulating microbubbles [8].

However, once the molecules have crossed the barrier, they have to diffuse in a highly constrained media, the extracellular space (ECS), to reach their targets [9]. Moreover, since the ECS architecture can change in case of pathologies [10, 11], the characterization of the hindrance experienced by molecules within the brain tissue is essential when designing new therapeutic compounds or diagnostic molecules for brain diseases. Diffusion constraints can be studied by estimating the ECS tortuosity (*λ*). This parameter compares the apparent diffusion coefficient (ADC) of a molecule within the complex architecture of the ECS to its diffusion coefficient in a free medium *D*_free_ [12]. Different strategies have been proposed to measure the ADC. The most widely used method is real-time iontophoresis [9, 13], using tetramethylammonium (TMA^+^) as a probe. This technique not only permits the *in vivo* characterization of the ADC but also, thanks to the small size of both detection electrodes and injection micropipette, proves minimally invasive, with consequent preservation of the integrity of the tissues. Its main drawback consists in the measurement relying on just one spatial point. More recently, diffusion-weighted magnetic resonance imaging (DW-MRI) has been proposed to noninvasively measure the ADC of water molecules in the brain [14, 15]. In comparison to the previous techniques, DW-MRI allows ADC measurements in deeper areas of the brain with a high (typically 2 mm isotropic) spatial resolution [16]. However, contrary to TMA^+^ and other techniques using labelled molecules that diffuse only across the ECS, DW-MRI detects water, which is also present in the intracellular compartment. To benefit from the advantages offered by MR in acquiring deep volumes of the brain, a new method has been recently introduced by our team, which allows us to detect molecular diffusion only in the ECS structure [17]. To do so, MR-CAs are directly injected into the brain tissue, and their diffusion is followed by acquisition of several longitudinal relaxation-time (*T*_1_) parametric maps. MR-CA concentration maps at different diffusion times are then calculated, and from these, the ADC is estimated. When compared to the typical diffusion-based MRI techniques, our method investigates larger areas of the brain with a higher spatial resolution (about 0.2 × 0.2 mm^2^ in plane and 1 mm in thickness). However, a major issue raised by this procedure consists in intracerebral injections inducing edema, which modifies the diffusion properties of brain tissue.

In the present study, we have used two noninvasive methods for the *in vivo* estimation of the ADC of different Gd chelates diffusing in the ECS after a FUS-induced BBB opening experiment. In both cases, contrast agent diffusion is recorded through dynamic acquisitions of MRI concentration maps. In the first method, the ADC evaluation is performed as in [17], e.g., by fitting a 2D Gaussian curve to the image intensity at different time points. However, diffusion of molecules delivered to the brain with the aid of FUS-induced BBB permeabilization depends on many factors, such as tissue and particle properties, as well as acoustic parameters. For this reason, as a second approach to estimate contrast agent diffusion, we introduce here the first diffusion model able to fully describe and predict at long space and time scales the result of a FUS-induced BBB opening experiment. This model takes into account acoustic pressure, particle size, blood pharmacokinetics, vascular permeability as a function of time, and diffusion process occurring in brain tissue.

Starting from ADC estimation performed with the help of both methods and the evaluation of *D*_free_ for all the compounds by means of *in vitro* experiments, it is possible to calculate tortuosities in the target region of rats' brains, to evaluate the effect of the selected BBB permeabilization protocol on the properties of brain tissue.

## 2. Materials and Methods

### 2.1. Experimental Procedures

**Figure 1:**
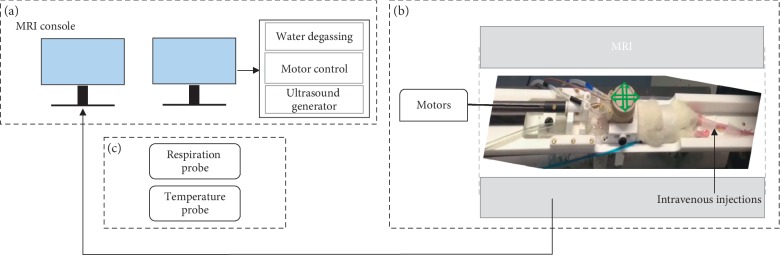
Experimental setup. (a) The MRI console and the computer driving the electronic for ultrasound, embedded in a tower composed by the water degassing system, the motor control, and the ultrasound generator. (b) The transducer and its electronic compatible with the MRI scanner. The transducer can move along the two perpendicular directions pictured by the green arrows. (c) The respiration and the temperature probes for real-time monitoring of the animal's vital signs.

All magnetic resonance acquisitions were performed by using a 7 T/90 mm Pharmascan scanner (Bruker, Ettlingen, Germany). The *in vitro* acquisitions have been performed by using a ^1^H transmit-receiver volume coil (Bruker). The *in vivo* experiments have been conducted by using a dedicated ultrasound single-loop radiofrequency coil [18], whose diameter was wide enough for the ultrasound beam to pass through it and for extensive displacement of the transducer above the rat's head. A heater device was used to keep temperature at the physiological value (37°C), as monitored by a temperature probe that was inserted inside the magnet (see Figure 1).

Three different gadolinium (Gd) chelates were studied: Dotarem® (Gd-DOTA, Guerbet, France), Gadovist® (Gd-DO3A-butrol, Bayer, Germany), and MultiHance® (Gd-BOPTA, Bracco, Italy). First, we assessed their longitudinal relaxivities (*r*_1_) at 7 T and 37°C, using phantoms made of bundles of tubes containing different contrast agent (CA) concentrations in 0.3% w/w agarose gel for each compound. For these phantoms, *T*_1_ values were measured by means of an inversion-recovery fast gradient echo (IR-FGE) sequence [17, 19] (echo time (*T*_E_)/repetition time (*T*_R1_) = 2.5/5 ms, 6 segments, 90 inversion times (TI) varying from 75 ms to 8975 ms, flip angle (FA) = 5°, matrix = 120 × 120 × 5 with resolution = 0.250 × 0.250 × 1.25 mm^3^, delay between the acquisitions of two segments (*T*_R2_) = 15000 ms, and number of averages (NA = 6)). Resulting relaxivities are summarized in Table 1. This table also includes the hydrodynamic diameter (*d*_H_) of each CA, measured by dynamic light scattering (DLS). DLS experiments were performed using a NanoZS equipement (Malvern, France) operating at an angle of 173°. For each Gd chelate, the DLS acquisitions were performed at 25°C by using concentrations of 0.5 M for MultiHance and Dotarem and of 1.0 M for Gadovist, e.g., without diluting the samples. We performed five different DLS measurements for each sample. The mean *d*_H_ and the standard deviation evaluated over the five measures are reported in Table 1.

Evaluations of the *D*_free_ of each compound were done with a method already presented in a previous work [17]. For each product, 10 *μ*L of a 5 mM solution was injected with a Hamilton syringe (diameter = 1 mm) into a tube filled with 0.3% w/w agarose gel. A stereotactic system was used to make the injection central and vertical with respect to the tube. The free diffusion of the CA was then dynamically followed by acquisition of five *T*_1_ parametric maps after injection (IR-FGE sequence with the following parameters: *T*_E_/*T*_R1_ = 2.5/5 ms, 6 segments, 60 TI from 88 ms to 5100 ms, FA = 5°, matrix = 128 × 104 × 14 with res = 0.225 × 0.225 × 1 mm^3^, TR_2_ = 9000 ms, NA = 1, and total duration = 12.5 min). A *T*_1_ map acquired before the injection was used as a reference.

The number of TI values has been chosen to ensure an accurate estimation of *T*_1_ values for a large range of *T*_1_. In particular, thanks to this sequence, we are able to detect Gd concentrations with a sensitivity threshold estimated around 2.5 *μ*M [17]. The spatial and temporal resolutions of this mapping sequence were set in order to ensure a sufficient space and time sampling of CA diffusion process. Further details about the optimization of this MRI sequence can be found in [17]. In all *T*_1_-parametric maps, all voxels with a *T*_1_ value larger than 5000 ms, that is, much larger than both the *T*_1_ of gray and white matter at 7 T [20], have been masked and considered as Not-a-Number.

The measurements of the ADC were performed *in vivo* on 9 Sprague Dawley male rats (3 rats/compound, 120–140 g, Janvier, Le Genest-Saint-Isle, France). Animal testing complied with the recommendations of the European Community (86/609/EEC) and French legislation (decree no. 87/848). The experimental setup is shown in Figure 1. The rats were anesthetized by means of 1.5–2% isoflurane in a mixture of air and oxygen, and their heads were chemically shaved to ensure a proper coupling with the ultrasound transducer. They were then placed in prone position in a cradle, integrating a stereotactic frame and a dedicated radiofrequency coil (Figure 1(b)). A custom build catheter (25G) was inserted into the caudal vein to perform injections from outside the MRI scanner. Temperature monitoring and breathing monitoring were performed using a rectal temperature probe and a respiration probe (Figure 1(c)), respectively.

A MR-compatible focalized transducer with 1.5 MHz central frequency (diameter: 25 mm, focal depth: 20 mm, focal spot dimensions: 1.1 mm in-plane, 6 mm thickness, Imasonic, France) was coupled to any animal's head via a balloon filled with degassed water. The transducer was mounted on a mobile stage, and its position could be tuned from outside the magnet by using MR-compatible motors (see Figure 1(b)). The movement of the motors and ultrasound parameters were controlled by a dedicated software (Thermoguide®, Image Guided Therapy, France) (Figure 1(a)). All acoustic pressures were estimated from previous calibration of the transducer, taking a skull transmission factor varying with animals' weight [21].

**Figure 2:**
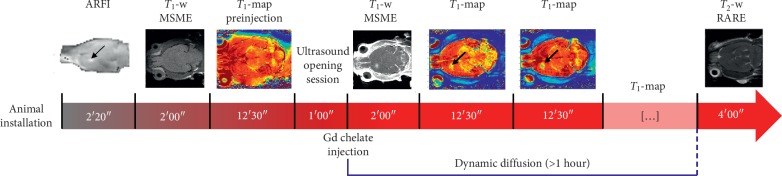
Experimental protocol for *in vivo* measurements: an ARFI sequence was used to detect the local acoustic intensity and choose the position of the BBB opening, indicated by the black arrow. This 2-minute acquisition was followed by a *T*_1_-weighted MSME sequence and by the first *T*_1_ map, acquired just before opening. One minute after the ultrasound opening session, the MRI contrast agent was injected. A *T*_1_-weighted image was acquired to evaluate the goodness of the opening procedure. About two minutes after the CA injection, the diffusion process was followed over more than 1 hour, by acquiring several *T*_1_ maps. At the end of each experimental session, a *T*_2_-weighted RARE image was acquired to evaluate damages, such as hemorrhages and edema, due to ultrasound. All the images shown in this figure refer to acquisitions performed by using Gadovist.

In Figure 2, the experimental protocol is shown. After rat installation, an acoustic radiation force imaging (ARFI) sequence [22, 23] was performed to localize the ultrasound focal point in rats' brains, consisting in a standard multislice multiecho sequence (MSME; *T*_E_/*T*_R_ = 28/1080 ms, matrix = 64 × 64 × 5, and res = 0.5 × 0.5 × 2 mm^3^) modified by the addition of two motion-sensitizing gradients (MSGs; duration of one MSG = 8 ms and duration of the ultrasound bursts = 4 ms). Knowing the current position of the focal spot, the transducer was moved using the motors so as to focalize ultrasound in the left striatum of the rats. This location has been chosen to ensure a high acoustic transmission through the skull as detailed in a recent work published by our team [21]. A second ARFI image was acquired to assess the good positioning of the ultrasound focal spot. *T*_1_-weighted (*T*_1_w) anatomical images were acquired, before the BBB opening, by using an MSME (*T*_E_/*T*_R_ = 8.3/300 ms, matrix dimension = 256 × 256 × 10, resolution of 0.125 × 0.125 × 1 mm^3^, and 3 averages). This was followed by a bolus injection of Sonovue® microbubbles (Bracco, Milan, Italy; 1.5 × 10^8^ bubbles/mL, 1.6 mL/kg, 3 s) via tail vein catheter, approximately 5 s before transcranial sonication (3 ms burst every 100 ms over a period of one minute; estimated focal acoustic pressure in the brain = 0.8 MPa). 30 seconds after the end of the ultrasound session, Gd chelates were intravenously injected via bolus (5 seconds, 0.5 M and 1.6 mL/kg for MultiHance and Dotarem; 1 M and 0.8 mL/kg for Gadovist). *T*_1_-weighted (*T*_1_w) images were acquired 30 seconds after the CA injection to verify the BBB disruption. Using the same IR-FGE sequence as the one used for *in vitro* diffusion, *T*_1_ parametric maps were acquired before and after sonication in order to dynamically follow the diffusion of the Gd chelates in the brain. At the end of each experimental session, a *T*_2_-weighted (*T*_2_w) image was acquired to verify the absence of any hemorrhage or edema due to ultrasound-induced BBB disruption. A rapid acquisition with relaxation enhancement (RARE) sequence was used with the following parameters: *T*_E_/*T*_R_ = 10/3800 ms, RARE factor = 8, and matrix = 128 × 128 × 32 with resolution = 0.225 × 0.225 × 0.5 mm^3^.

### 2.2. Data Analysis

From *T*_1_ maps, the corresponding concentration maps were calculated using the following relationship between the longitudinal relaxation rates, 1/*T*_1_, and the Gd-chelate concentrations, [CA] [24]:

(1) $$\begin{array}{c} \frac{1}{T_{1}}=\frac{1}{T_{1,0}}+r_{1}\cdot \left[\text{CA}\right], \end{array}$$

where 1/*T*_1,0_ is the relaxation rate of the sample without CA, i.e., before the injection. From this equation, CA concentration maps were then obtained. All voxel values in the *T*_1_ or *T*_1,0_ maps larger than 5000 ms were considered as Not-a-Number in the CA maps. These voxels were not considered in the CA-diffusion analysis.

In all cases (both for *in vivo* and *in vitro* acquisitions), we have assigned to each CA map the time elapsed between the CA injection (in agarose gel or in the caudal vein for the *in vitro* and the *in vivo* acquisitions, respectively) and the beginning of the CA-map acquisition sequence.

To evaluate the *D*_free_ value of injected molecules, the following bidimensional Gaussian function was fitted to concentration-map data for each time point after the CA injection [17, 25, 26]:

(2) $$\begin{array}{c} \left[\text{CA}\left(x,y\right)\right]=Ae^{\left(-a{\left(x-x0\right)}^{2}-2b\left(x-x0\right)\left(y-y0\right)-c{\left(y-y0\right)}^{2}\right)}, \end{array}$$

where *A* is the Gaussian amplitude and (*x*_0_, *y*_0_) are the coordinates of its center along the absolute axes (*x*, *y*). *a*, *b*, and *c* are functions depending on the Gaussian widths (*σ*_*X*_ and *σ*_*Y*_) along its main axes (*X* and *Y*) and on the angle *θ* between (*X*, *Y*) and (*x*, *y*):

(3) $$\begin{array}{c} a=\frac{\cos^{2}\left(\theta \right)}{2\sigma_{X}^{2}}+\frac{\sin^{2}\left(\theta \right)}{2\sigma_{Y}^{2}},\\ b=-\frac{\sin\left(2\theta \right)}{4\sigma_{X}^{2}}+\frac{\sin\left(2\theta \right)}{4\sigma_{Y}^{2}},\\ c=\frac{\sin^{2}\left(\theta \right)}{2\sigma_{X}^{2}}+\frac{\cos^{2}\left(\theta \right)}{2\sigma_{Y}^{2}}. \end{array}$$

The regression algorithm used to fit the data with Gaussian functions is the Levenberg–Marquardt algorithm [27], available in the GSL, GNU Scientific Library (https://www.gnu.org/software/gsl/doc/html/nls.html). In particular, we used the version of this algorithm implemented in the scaled LMDER routine in MINPACK, written by Jorge J. More, Burton S. Garbow, and Kenneth E. Hillstrom (https://people.sc.fsu.edu/∼jburkardt/f_src/minpack/minpack.html).

Defining *σ*_*X*_^2^ and *σ*_*Y*_^2^ as the molecular mean square displacements along *X* and *Y*, the diffusion coefficients along these axes, *D*_free,*X*_ and *D*_free,*Y*_, are given by Fick's law:

(4) $$\begin{array}{c} D_{\text{free,}X\text{,}Y}=\frac{\sigma_{X,Y}^{2}}{2t}, \end{array}$$

where *t* is the instant time after injection, i.e., the diffusion time. *D*_free_ values were then calculated as the mean value of *D*_free,*X*_ and *D*_free,*Y*_ components:

(5) $$\begin{array}{c} D_{\text{free}}=\frac{\left(D_{\text{free},X}+D_{\text{free},Y}\right)}{2}. \end{array}$$

The first method used to evaluate the ADC consisted in placing a mask surrounding the disruption site in CA maps, to which the same Gaussian fitting procedure was applied. For any compound, the ADC was estimated in any rat's striatum as the average:

(6) $$\begin{array}{c} \text{ADC}=\frac{\left({\text{ADC}}_{X}+{\text{ADC}}_{Y}\right)}{2}. \end{array}$$

The second ADC estimation took into account how the BBB permeabilization changes after the ultrasound application, together with CA pharmacokinetics after injection. A homemade MATLAB code was used to simulate CA diffusion within the ECS after the BBB opening.

The code comprises the following components:

- A source function *S* (*x*, *y*, *z*, *t*), describing the contrast agents that move from the blood to the brain, was modeled as

(7) $$\begin{array}{c} S\left(x,y,z,t\right)=\alpha \cdot Q_{\text{CA}}\left(x,y,z,t\right)\cdot {\text{CA}}_{\text{blood}}\left(t\right), \end{array}$$

  where *α* is a proportionality constant, requiring a first guess on its value, *Q*_CA_(*x*, *y*, *z*, *t*) is the amount of CA crossing the BBB [28], and CA_blood_(*t*) describes CA pharmacokinetics. For a Gd chelate of hydrodynamic diameter (*d*_H_), *Q*_CA_(*x*, *y*, *z*, *t*) is defined as [28, 29]

(8) $$\begin{array}{c} Q_{\text{CA}}\left(x,y,z,t\right)∼\frac{\sigma_{0}^{2}e^{-2kt}}{d_{\text{H}}}\cdot \left(\sqrt{\frac{\pi }{2}}\left(1-\text{erf}\left(\frac{d_{\text{H}}}{\sqrt{2}\sigma_{0}\left(x,y,z\right)e^{-kt}}\right)\right)+\frac{d_{\text{H}}}{\sigma_{0}\left(x,y,z\right)e^{-kt}}e^{-\;\left(d_{\text{H}}^{2}/\left(2\sigma_{0}^{2}\left(x,y,z\right)e^{-2kt}\right)\right)}\;\right), \end{array}$$

  where *σ*_0_ is the standard deviation of the distribution of the gap sizes generated in the BBB by ultrasound and *k* is the BBB closure rate (*k* = 1.54e^−5^·s^−1^). Since it has been demonstrated that blood-brain barrier disruption is characterized by a mechanical index (MI), which is linearly dependent on the effective acoustic pressure (*P*_ex_) [30], we considered the same dependence for *σ*_0_. In particular, according to the work published by Marty et al. in 2013 [28], we applied the relationship *σ*_0_ = 2.1·*P*_ex_. Starting from the simulated acoustic pressure map, we obtained the *σ*_0_(*x*, *y*, *z*) distribution.
  The kinetic term in equation (7) can be expressed by

(9) $$\begin{array}{c} {\text{CA}}_{\text{blood}}\left(t\right)={\text{CA}}_{\text{inj}}\cdot \;\exp\left(\frac{-t}{b}\right), \end{array}$$

  since our time resolution in the acquisition of CA maps (12.5 min) allows for just considering the wash out of CAs, in obedience to Tofts' two-compartment kinetic model [31]. CA_blood_(*t*) depends on the injected CA concentration (CA_inj_) and on its clearance rate from the blood, *b*. CA_inj_ was estimated for each animal by taking into account its weight and an average blood volume of 6.86 mL/100 g [32], while *b* was fixed at 25 minutes [33].
(ii) Introducing the source term *S*(*x*, *y*, *z*, *t*) into Fick's second law, the evolution of CA-concentration long time was found by simulating the equation

(10) $$\begin{array}{c} \frac{\partial \left[\text{CA}\right]\left(x,y,z,\;t\right)}{\partial t}={\text{ADC}}_{x}\cdot \frac{\partial^{2}\left[\text{CA}\right]\left(x,y,z,\;t\right)}{\partial x^{2}}+{\text{ADC}}_{y}\cdot \frac{\partial^{2}\left[\text{CA}\right]\left(x,y,z,\;t\right)}{\partial y^{2}}+{\text{ADC}}_{z}\cdot \frac{\partial^{2}\left[\text{CA}\right]\left(x,y,z,\;t\right)}{\partial z^{2}}+S\left(x,y,z,\;t\right), \end{array}$$

  for a temporal and spatial resolution higher than those characterizing [CA] maps. Specifically, an isotropic spatial resolution (d*x*, d*y,* d*z*) equal to 0.125 *μ*m was selected, while the temporal step d*t* was set at 5 s. While *α* has been guessed, the initial ADC values used for simulations were chosen from the equation

(11) $$\begin{array}{c} \lambda =\sqrt{\frac{D_{\text{free}}}{\text{ADC}}}\;, \end{array}$$

  starting from tortuosity values of the target region of the brain recovered from the literature [34] and molecular *D*_free_ values retrieved from our experiments.
(iii) The simulated CA volume was downsampled in space and time to the MRI acquisition resolution and then coregistered to the experimental three-dimensional [CA] distribution in the CA-concentration maps.

Due the large focal-spot length (∼6 mm), CA concentration can be considered as constant along this direction (called *z*) for all the slices taken in account. This makes the CA gradient negligible along *z*, as well as the related diffusional process (see Figures [Supplementary-material supplementary-material-1] and [Supplementary-material supplementary-material-1] in the Supplementary Materials). For this reason, the previous equation can be considered as reasonably describing the following bidimensional dynamics:

(12) $$\begin{array}{c} \frac{\partial \left[\text{CA}\right]\left(x,y,\;t\right)}{\partial t}={\text{ADC}}_{x}\cdot \frac{\partial^{2}\left[\text{CA}\right]\left(x,y,\;t\right)}{\partial x^{2}}+{\text{ADC}}_{y}\cdot \frac{\partial^{2}\left[\text{CA}\right]\left(x,y,\;t\right)}{\partial y^{2}}+S\left(x,y,t\right). \end{array}$$

This last equation was integrated in order to estimate [CA](*x*, *y*, *t*). Through a cumulative fit including the experimental CA maps for the central slice, the ADC components along *x* and *y* and the proportionality constant *α* were found. Different ADCs around the value suggested by equation (11) were simulated until the fit algorithm converged.

To evaluate the quality of the experimental approach chosen to mimic molecular free diffusion (i.e., the injection of the compound in 0.3% w/w of agarose gel), it is worth estimating the hydrodynamic diameter of the molecules, using the Stokes–Einstein equation:

(13) $$\begin{array}{c} d_{\text{H}}=\frac{kT}{3\pi \eta D_{\text{free}}}, \end{array}$$

where *k* = 1.38·10^−23^ Pa·m^3^·K^−1^ is the Boltzmann constant, *T* is the temperature in Kelvin degrees, and *η* is the viscosity of the agar gel (6.92·10^−4^ Pa·s).

From the mean ADC recovered through the two aforementioned methods, the tortuosity values were estimated with the help of equation (11).

## 3. Results

**Figure 3:**
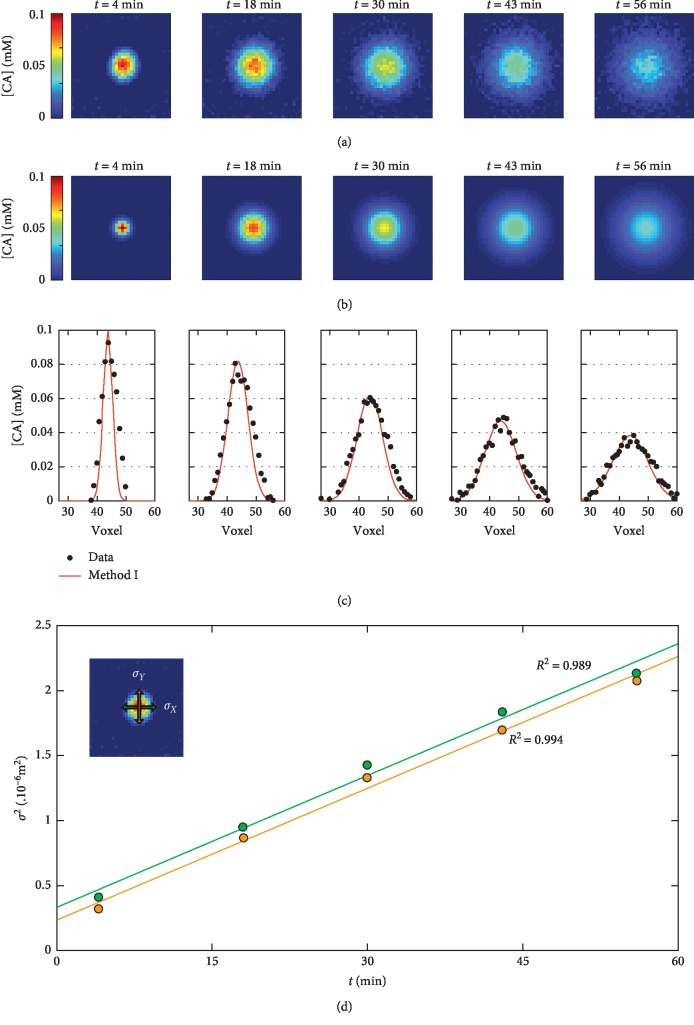
*In vitro* diffusion of MultiHance. (a) Concentration maps acquired during 1 hour after the injection of 200 *μ*L of the 5 mM contrast agent in a phantom made of 0.3% w/w agarose gel. The time reported above each CA map refers to the time elapsed since the CA injection. (b) Concentration maps obtained by fitting the maps shown in (a) through equation (2), for each time point. (c) Shows a profile of the [CA] values (black dots), in the central rows on (a) and their corresponding fit (red line) from (b). These curves are shown for each time point. In (d), the trends of the square values of the Gaussian widths are shown as a function of the diffusion time. In green and orange are pictured the experimental data and the linear fits *σ*_*X*,*Y*_^2^=*D*_*X*,*Y*,vitro_ · 2*t* for *σ*_*X*_^2^ and *σ*_*Y*_^2^, respectively.

Figure 3 shows an example of *in vitro* diffusion data and their analysis. Concentration maps (Figure 3(a)) were acquired 4 to 56 minutes after the injection of MultiHance. These data were fitted by means of the bidimensional Gaussian function reported in equation (2). The simulated Gaussian distributions resulting from the fit are shown in Figure 3(b). Taking into account the voxel values in the central row of the Gaussian spots pictured in Figures 3(a) and 3(b), it is possible to assess the quality of the fit, as illustrated in Figure 3(c), where the black dots represent the data and the red curve their Gaussian fit.

Fick's law (equation (4)) was used to fit the squares of the fitted Gaussian widths (*σ*_*x*_ and *σ*_*y*_) as a function of the diffusion time, in order to obtain an estimation of *D*_free,*X*_ and *D*_free,*Y*_ (Figure 3(d)). The *D*_free_ values found for each compound are given by the average of the two components and are summarized in Table 1.

**Figure 4:**
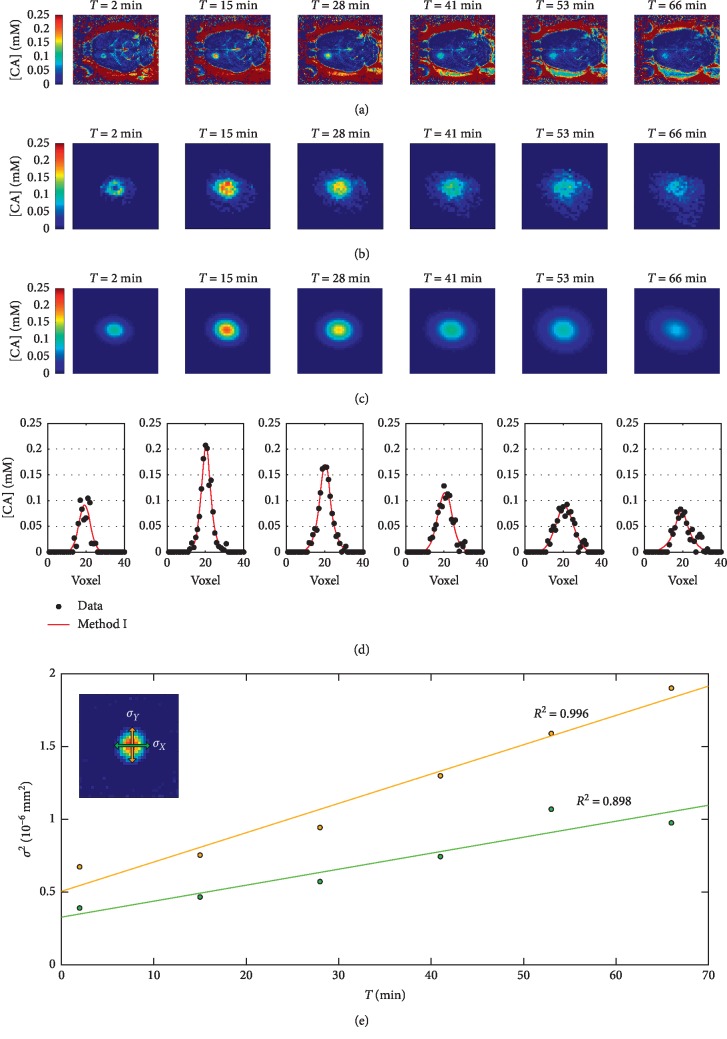
*In vivo* experiments performed with Dotarem where the BBB has been opened in the left striatum. Figure (a) shows the concentration maps acquired between 2 and 66 minutes after the injection. The masked maps used to perform the Gaussian fits are shown in row (b), while the Gaussian surfaces obtained with the fit are pictured in (c). By comparing, through a two-sample Kolmogorov–Smirnov test, the data shown in (c) with the respective Gaussian profiles at each time point, we obtained *p* values equal to 5.6*e* − 4; 0.258, 0.258, 0.440, and 0.2581. (d) shows Gaussian profiles (red line), fitting the [CA] values (black dots), in the rows going through the centers of the spots in (b). The squares of the Gaussian widths, *σ*_*X*_ and *σ*_*Y*_, are plotted over the diffusion time with the linear fit *σ*^2^_*X,Y*_ = *D*_*X*,*Y*,vivo_ · 2t in (e), where the green and the orange colors refer to *σ*_*X*_^2^ and *σ*_*Y*_^2^, respectively.

The ADCs were estimated by analyzing *in vivo* concentration maps, as the ones shown in the upper panel of Figure 4. Specifically, these maps were acquired 2 to 84 minutes after bolus injection of Dotarem. Prior to compute Gaussian fits on concentration maps, a mask including only the BBB disruption site was applied (Figure 4(b)). The first method for ADC evaluation consists in fitting 2D Gaussian functions to such maps. The resulting distributions are shown in Figure 4(c).

As for the *in vitro* measurements, the overlapping between data and fit curve is shown (see Figure 4(d)). By comparing, through a two-sample Kolmogorov–Smirnov test, the data shown in Figure 4(c) with the respective Gaussian profiles at each time point, we obtained *p* values equal to 5.6*e* − 4; 0.258, 0.258, 0.440, and 0.2581, meaning that only at the first time point the Gaussian fit results to be different from the data. We also evaluated the ADC values without taking into account the first time point. However, since the values obtained with and without the first time point varied less than the error estimated by the respective linear fits and less than the variations inside the *n* = 3 rat pools, we also considered the first time point to estimate the ADCs.

The temporal evolution of the squared Gaussian widths is shown in Figure 4(e), together with their fits by Fick's Law. Starting from ADC_*X*_ and ADC_*Y*_ values, the ADC in each rat's striatum was found. By average over the entire set of rats, the mean ADCs, reported in Table 2, were estimated, as well as brain tortuosity, *λ*_I_.

The second method proposed to evaluate brain diffusional properties is based on a model taking in account both the temporal changes in BBB permeabilization after ultrasound application and CA blood pharmacokinetics.

**Figure 5:**
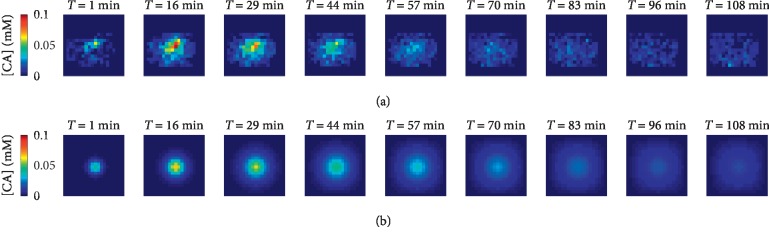
Results obtained by using the second method of evaluation of the ADCs to investigate the delivery of MultiHance within one rat brain. In (a), we show the masked CA acquired for more than 1 hour, after the BBB opening induced by ultrasound. (b) shows the results of our best fit simulation. In particular, the central slice showing the maximum CA concentration is pictured along the diffusion time. The times reported above each CA maps refer to the times elapsed after the injection of the compound.

Figure 5 shows an example of CA distributions inside the brain, obtained by fitting this model to experimental concentration maps obtained by diffusion measurements on Multihance.

Once again, Figure 5(a) reports the masked concentration maps used to evaluate brain tortuosity, while the maps in Figure 5(b) are obtained via model. The ADCs estimated by average of model results obtained for each compound are shown in Table 2 (ADC_II_). In the same table, the values obtained for the proportionality constant *α* of the source term are included. Entering *D*_free_ and ADC_II_ values found by this second approach in equation (11), brain tortuosity is once again retrieved (*λ*_II_ in Table 2).

**Figure 6:**
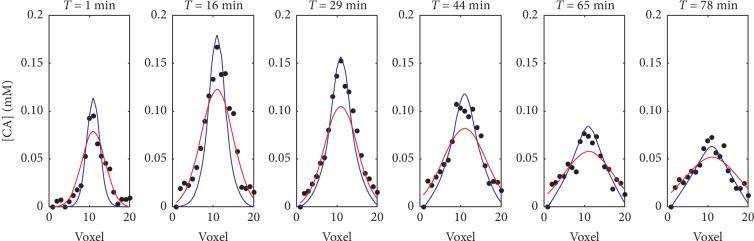
Example of CA distributions over time, after the CA injection. These data refer to the diffusion of Gadovist and are pictured with the black dots. In red, the Gaussian fits are shown (method I of analysis), whereas in blue are shown the distributions profiles obtained with method II, e.g., our mathematical model. By comparing, through a two-sample Kolmogorov–Smirnov test, the data shown in figure with the respective Gaussian and simulated profiles at each time point, we obtained *p* values equal to 0.985, 0.374, 0.147, 0.047, 0.147, and 0.047 for method I and equal to 0.675, 0.675, 0.736, 0.736, 0.736, and 0.736 for method II.

For the sake of comparison, in Figure 6, the distribution profiles extracted from the centers of [CA] maps are shown, as previously done in Figure 4(d). This dataset refers to an experiment on Gadovist with black dots representing experimental data and [CA] red and blue profiles representing theoretical data obtained from the first and second method, respectively. By comparing, through a two-sample Kolmogorov–Smirnov test, the data with the simulated and the Gaussian profiles, we obtained, at different time points, *p* values equal to 0.985, 0.374, 0.147, 0.047, 0.147, and 0.047 for method I and equal to 0.675, 0.675, 0.736, 0.736, 0.736, and 0.736 for method II.

These results show that method II allows for obtaining distribution shapes that are more similar to data at all the time points than Gaussian fits in method I.

## 4. Discussion

This work introduces two new methods suitable for the *in vivo* characterization of molecular diffusion processes taking place in the ECS after transient BBB permeabilization with low-intensity focused ultrasound in order to deliver MR-contrast agents to the brain. We used MRI to record MR-CA diffusion. By measuring *D*_Free_ (free-medium diffusion) and ADC values within the ECS, brain tissue tortuosity was calculated in order to have information on brain architecture.

To assess the quality of the experimental approach chosen to evaluate molecular free diffusion, it is worth comparing the hydrodynamic diameter of the molecules, *d*_H_(S-E), obtained through equation (13), to the ones found by using DLS. As can be noticed from Table 1, the hydrodynamic diameter found through these two methods agree, which means that the diffusion of the compounds in 0.3% w/w of agarose gel can be considered as free. In addition, *D*_free_ values in Table 1 can be compared to the analogous ones already published in the literature. Specifically, Marty et al. [17] have found the same *D*_free_ for Dotarem, whereas Thorne and Nicholson [35] have estimated a free diffusion coefficient equal to (2.22 ± 0.16)·10^−10^ m^2^/s for a molecule with hydrodynamic diameter of 2.95 ± 0.02 nm, which is comparable to one that was found for a slightly smaller molecule of MultiHance (*d*_H_ = 2.3 ± 0.1 nm and *D*_free_ = (2.8 ± 0.2) · 10^−10^ m^2^/s).

Table 2 shows that, irrespective of the applied method, ADC values scale correctly with molecular size, decreasing at increasing *d*_H_ (ADC_Dotarem_ > ADC_Gadovist_ > ADC_MultiHance_), as expected from comparison to the literature [35]. Furthermore, all ADC values are smaller than their associated *D*_free_, which confirms the hindrance experienced by diffusion across the ECS.

Tortuosities obtained by method I and II (*λ*_I_ and *λ*_II_) are compared to those appearing in the literature in order to assess the goodness of ADC estimation.

*λ*_I_ and *λ*_II_ obtained for the different molecules turn out constant, which agrees with the literature. Indeed, all of our test molecules have a hydrodynamic diameter ten times smaller than the intracellular gap *d*, which is typically comprised between 20 and 64 nm in healthy rats' brains [35, 36].

In this case, the stationary wall-drag effect, expected for larger molecules by virtue of viscosity theory, affects neither molecular diffusion [36] nor tortuosity, whose value only depends on the ECS structure and not on the size of the diffusion probes.

### 4.1. Limitations and Future Perspectives

In the present work, both the methods used to estimate the molecular apparent diffusion coefficients are based on a protocol validated by our team in 2013 [17], e.g., the dynamic acquisitions of CA-concentration maps through an IR-FGE MRI sequence. Although this sequence has been accurately tuned to be sensitive to a large range of CA concentrations and to have a sufficiently high temporal and spatial resolution to record molecular diffusion, further work is needed to improve such resolutions. For example, a suitable way to increase the speed of the MRI sequence currently used is by using compressed sensing MRI techniques [37]. Doing so, we expect to reduce the acquisition time and therefore to get access to diffusion data of MRI contrast agents at high temporal resolution.

The second limitation of our experimental approach is related to the possibility to evaluate CA diffusion only in two dimensions. Indeed, our method allows us to estimate the transversal components (*x* and *y*) of the ADC but not to evaluate CA diffusion processes along *z*-axis. This is due to the gradient concentration and to the relatively low spatial resolution in this direction. In order to improve our delivery method and to be more sensitive to Gd concentration gradients along *z*-axis, future experiments can be performed by using multielement transducer to produce a controlled steering of the ultrasound beam in the *z* direction (see [Supplementary-material supplementary-material-1] in the Supplementary Materials). With this steering approach, it will be possible to permeabilize the BBB in a smaller region of the brain. In addition, by improving the spatial resolution in *z* of the concentration maps, it will be then possible to characterize the particle diffusion also along this direction.

Another limitation of our work concerns the capability of method II to fully predict the amount of particles getting in the brain after a FUS-induced BBB opening experiment. Indeed, from a qualitative point of view, one can expect the inclusion of the source term to provide a better data description when the blood-to-ECS flux is larger, i.e., for CAs of smaller size, since the Q_CA_ expression is a monotonically decreasing function with the molecular hydrodynamic diameter, *d*_H_. However, the amount of particles getting in the brain after a FUS-induced BBB permeabilization is dependent from many factors, some of them being difficult to precisely control. For example, if the coupling of the water balloon between the transducer and the head, or if the position of the transducer, slightly changes between two experiments, the transmitted acoustic power could vary inside the brain and, consequently, the amounts of particles delivered to brain tissue [21, 38]. McDannolds et al. [39] have recently shown that even the level of oxygen used as a carrier gas for anesthesia during the experiments can change microbubble activity and BBB disruption. All these aspects, varying among experiments, change the value of the constant of proportionality α. For this reason, in order to use our model to simulate an experimental outcome, the simulations need to be performed by varying *α* between 0 (e.g., the worst-case scenario corresponding to a failure of the experiment) and 0.07 (e.g., the maximum value of *α* found in this work).

### 4.2. Comparison between the Two ADC Estimation Methods

The first method consists in fitting Gaussian distributions to CA-map data in the brain region where diffusion occurs. From this fit, the molecular square displacements, and so their ADC, can be evaluated. This kind of postprocessing is already accepted in the literature [16], although originally applied to CA diffusion patterns acquired after intracerebral injection of compounds. However, this method presents some limitations. The first one concerns the application of this fit to CA maps with low signal-to-noise ratio (SNR).

In particular, we define the SNR in each slice of the CA maps, as the ratio between the maximum CA delivered in the slice and the standard deviation in a region (20 voxels × 20 voxels) located in the contralateral hemisphere. The Gaussian fit overestimates the distribution widths for SNR smaller than 10. This is the case, for example, of the acquisition shown in Figure 6. The errors committed by method I on the estimation of the distributions widths are confirmed by the *p* values obtained when comparing the Gaussian profiles to the respective data points, through a two-sample Kolmogorov–Smirnov test. Indeed, the *p* values resulted to be smaller than 0.05 at two time points. The same issue does not affect ADC estimations when the compounds are intracerebral injected, as in [17]. Indeed, in this latter case, the SNR is higher than the one obtained through BBB opening since the CA concentration diffusing within the ECS is 100 times larger than the CA delivered through BBB-opening.

On the other hand, when method II is applied to analyze the same dataset, it is possible to obtain particle distributions more similar to the experimental ones, as confirmed by the *p* values larger than 0.05, resulting from the same kind of statistical test.

In addition, to fit the data through the first method, we use the version of Levenberg–Marquardt algorithm implemented in the scaled LMDER routine in MINPACK [27]. This scaled LMDER routine makes use of both the function and its derivative, so it could explain why in some cases, as the one shown in Figure 6, the main differences between the data and the respective Gaussian fit can be found near the peak.

With respect to the first method, the second ADC estimation method presented in this work is based on a diffusion model that includes a source term. The source term describes the flux from the blood to the ECS only, which is appropriate if the two pools have a large concentration difference. This approximation can be quantitatively justified. Indeed, the CA concentration injected in the blood system is around 3 mM, while, as can be noticed from Figures 4–6, the maximum CA delivered in the brain is estimated to be approximately 100 times smaller. In addition, the CA concentration in blood is much higher than the ECS concentration, during the duration of whole of the experiments (about 1 hour) (see [Supplementary-material supplementary-material-1] in Supplementary Materials).

Another possible way to compare the two methods is to compare the different tortuosity values, *λ*_I_ and *λ*_II,_ shown in Table 2. It has been recently shown with histology that low-intensity pulsed ultrasound could be used to transiently enlarge the ECS width [40]. In particular, by estimating the overall volume of distribution of different nanoparticles, Frenkel et al. found an enhanced volume of 36% in average. The volume where particles diffuse in ECS is characterized by the volume fraction *υ* = *V*_ECS_/*V*_T_, defined as the ratio between the volume of ECS (*V*_ECS_) and the volume of the whole tissue measured in a small region of the brain (*V*_T_) (Sykova, Physiol Rev. 2008). In healthy brain tissue, the ECS volume fraction *υ* is estimated around 0.20. However, by considering the study proposed by Frenkel et al. [40], the volume fraction enlarges of 36% after FUS application, leading to a volume fraction of *υ* = 0.27. Since the relationship between the tortuosity value, *λ*, and *υ* is the following as given by [41]:

(14) $$\begin{array}{c} \lambda =\sqrt{2-\upsilon }, \end{array}$$

and the expected value of brain tortuosity after a FUS-induced BBB permeabilization experiment is equal to 1.32, e.g., more similar to the values obtained through method II than the ones estimated through the Gaussian fit.

## 5. Conclusions

In this study, we used two methods to characterize the contrast agent bidimensional diffusion within the brain after ultrasound-induced BBB opening. These techniques allow to investigate macromolecules biodistribution within the ECS with a slow time scale, suitable for the study of cellular uptake and transport, as well as of the potential clearance processes related to bulk flow or glymphatic pathway. Although it is well known that focused ultrasound combined with microbubbles permits to transiently and noninvasively break tight junctions, locally increasing the BBB permeabilization and so promoting drug delivery into the brain [8, 28, 42–44], so far no study has been performed to fully characterize, on a macroscopic space and time scale, the distribution of a compound when it enters the brain.

By using a motorized and MR-compatible ultrasound system, we were able to target the right striatum of 9 rats in a very precise and reproducible manner, in order to study diffusion processes in a specific area of the brain. Three commercially available MR-CAs were tested (Dotarem®, Gd-DOTA; Gadovist®, Gd-DO3A-butrol; MultiHance®, Gd-BOPTA). Their diffusion from the BBB-disruption site was followed by acquisition of several CA maps within 1 hour from application of ultrasound. The tested compounds are characterized by a similar hydrodynamic diameter (about 1–2 nm), which resulted in a similar hindering of diffusion in the ECS. Since the CA distribution depends on the diffusion properties of brain tissue, we have evaluated its tortuosity, a parameter comparing molecular ADC inside the tissue to its free-diffusion counterpart in a media without obstacles. The methods proposed here to estimate *λ* are both based on data processing of MR-CA maps. The first approach does not describe the dependence of molecular diffusion neither on fundamental biological aspects nor on the specific protocol used to permeabilize the BBB.

For this reason, we have presented a mathematical model able to fully predict time evolution of CA distributions within the brain after BBB permeabilization induced by FUS. Our model takes into account different biological features concerning the BBB-opening mechanism, such as the gap distribution between endothelial cells, in turn depending on the effective acoustic pressure transmitted through the skull and the shape of the focal spot, the BBB closure rate, and the CA concentration in blood after bolus injection and its physiological rate of clearance. The match with the experimental data allows us to introduce this approach as a new tool to successfully predict and plan drug distribution after a BBB-opening experiment, for any particle size and acoustic parameter, in all brain regions.

## Figures and Tables

**Table 1:** Table reporting the characteristics of the three contrast agents: longitudinal relaxivity *r*_1_ (s^−1 ^mM^−1^) measured at 7 T and 37°C; hydrodynamic diameter found from both DLS measurements, *d*_H_(DLS) and by using the Stokes–Einstein equation, *d*_H_(S-E); free diffusion (*D*_free_) of the molecules. Standard deviations (SD) are shown in bracket. The SD of the *D*_free_ values has been calculated by averaging the error estimated on both *D*_free,*X*_ and *D*_free,*Y*_ components when fitting the Gaussian widths through equation (4).

Compound	Number of phantoms	*r*_1_ (s^−1 ^mM^−1^)	*d*_H_ (DLS) (nm)	*d*_H_ (S-E) (nm)	*D*_free_ (10^−10^ m^2^/s)
Dotarem	1	4.7 (0.2)	1.6 (0.1)	1.5 (0.1)	4.5 (0.2)
Gadovist	1	5.5 (0.3)	1.8 (0.1)	1.7 (0.1)	3.9 (0.2)
MultiHance	1	6.9 (0.3)	2.3 (0.1)	2.3 (0.1)	2.8 (0.2)

**Table 2:** The ADC and the *λ* values found with both methods are reported, where the index I refers to the 2D gaussian fit method. The ADC_II_ and the *λ*_II_ are the results obtained from the new model introduced in this work, mimicking all the physiological processes occurring during an experiment of FUS-induced blood-brain barrier opening for drug delivery (see Section 2.2). The parameter *α* is a proportionality factor used in the method II. Standard deviations are shown in bracket.

Compound	Number of rats	ADC_I_ (10^−10^ m^2^/s)	ADC_II_ (10^−10^ m^2^/s)	*α* (10^−2^ a.u.)	*λ*_I_	*λ*_II_
Dotarem	3	1.8 (0.6)	3.2 (0.4)	3.6 (0.5)	1.6 (0.2)	1.2 (0.1)
Gadovist	3	1.5 (0.1)	2.9 (0.3)	2.2 (0.2)	1.5 (0.5)	1.2 (0.1)
MultiHance	3	1.3 (0.3)	1.8 (0.5)	4.5 (3.5)	1.5 (0.2)	1.3 (0.2)

## Data Availability

The MRI data used to support the findings of this study are available from the corresponding author upon request.

## Abbreviations

BBB: Blood-brain barrier
US: Ultrasound
FUS: Focused ultrasound
CA: Contrast agents
CA map: CA concentration map
ADC: Apparent diffusion coefficient
ECS: Extracellular space.

## Supplementary Materials

Supplementary Materials Supplementary Figure 1: (A) acoustic pressure field at 1.5 MHz. The pressure field is normalized by the maximum pressure (obtained at the focal spot). The acoustic pressure field shown on the right has been rescaled to the spatial resolution of the concentration maps. (B) Axial and sagittal views of the Gd-concentration maps: it can be noticed that the focal spot dimension along *z*-axis is comparable to the thickness of the rat brain in the area where the BBB was permeabilized. Moreover, the spatial resolution in this direction is much lower than the in-plane resolution (around 4.4 times). This significantly lower resolution along *z*-axis does not allow to precisely quantify the variations of CA concentration in this direction. Supplementary Figure 2: left: sagittal views of the Gd-concentration maps; right: concentration profiles extrapolated from the center of the BBB opening site. These profiles show that the Gd concentration changes only slightly with the *z* position. This is due to the low resolution of the Gd-concentration map along *z* and also to the dimensions of the focal spot along this direction. Supplementary Figure 3: acoustic pressure fields at 1.5 MHz for the concave transducer (*F*/*D* = 0.8) without steering (left) and with a 2.5 mm steering toward the transducer (right). Both pressure fields are normalized by the maximum pressure obtained at the focal spot in case of the absence of steering. With a 2.5 mm steering toward the transducer, the volume of the focal spot is decreased by 20% and the maximum pressure is increased by 10% compared to the experiment without steering. Supplementary Figure 4: comparison between the CA concentration in blood (picture in blue) and the maximum CA delivered during a BBB opening experiment (in red). In particular, experimental points refer to the data shown in Figure 4, while the trend of CA_blood_ along time, *t*, has been derived through the equation CA_blood_(*t*) = CA_inj·_exp(−*t*/*b*), with *b* = 25 minutes (Aime and Caravan, JMRI, 2009).
