# Supplementary material for: Empirical and Theoretical Characterization of the Diffusion Process of Different Gadolinium-Based Nanoparticles within the Brain Tissue after Ultrasound-Induced Permeabilization of the Blood-Brain Barrier
Source: Contrast Media Mol Imaging. 2019 Dec 1;2019:6341545. doi: 10.1155/2019/6341545 (PMC6914891; doi:10.1155/2019/6341545)
Supplement: Supplementary Materials — Supplementary Figure 1: (A) acoustic pressure field at 1.5 MHz. The pressure field is normalized by the maximum pressure (obtained at the focal spot). The acoustic pressure field shown on the right has been rescaled to the spatial resolution of the concentration maps. (B) Axial and sagittal views of the Gd-concentration maps: it can be noticed that the focal spot dimension along z-axis is comparable to the thickness of the rat brain in the area where the BBB was permeabilized. Moreover, the spatial resolution in this direction is much lower than the in-plane resolution (around 4.4 times). This significantly lower resolution along z-axis does not allow to precisely quantify the variations of CA concentration in this direction. Supplementary Figure 2: left: sagittal views of the Gd-concentration maps; right: concentration profiles extrapolated from the center of the BBB opening site. These profiles show that the Gd concentration changes only slightly with the z position. This is due to the low resolution of the Gd-concentration map along z and also to the dimensions of the focal spot along this direction. Supplementary Figure 3: acoustic pressure fields at 1.5 MHz for the concave transducer (F/D = 0.8) without steering (left) and with a 2.5 mm steering toward the transducer (right). Both pressure fields are normalized by the maximum pressure obtained at the focal spot in case of the absence of steering. With a 2.5 mm steering toward the transducer, the volume of the focal spot is decreased by 20% and the maximum pressure is increased by 10% compared to the experiment without steering. Supplementary Figure 4: comparison between the CA concentration in blood (picture in blue) and the maximum CA delivered during a BBB opening experiment (in red). In particular, experimental points refer to the data shown in Figure 4, while the trend of CAblood along time, t, has been derived through the equation CAblood(t) = CAinj·exp(−t/b), with b = 25 minutes (Aime and Caravan, JMRI, 2 [file 6341545.f1.docx]

Empirical and theoretical characterization of the diffusion process of different Gadolinium-based nanoparticles within the brain tissue after ultrasound-induced permeabilization of the Blood-Brain Barrier

**Supplementary Materials**


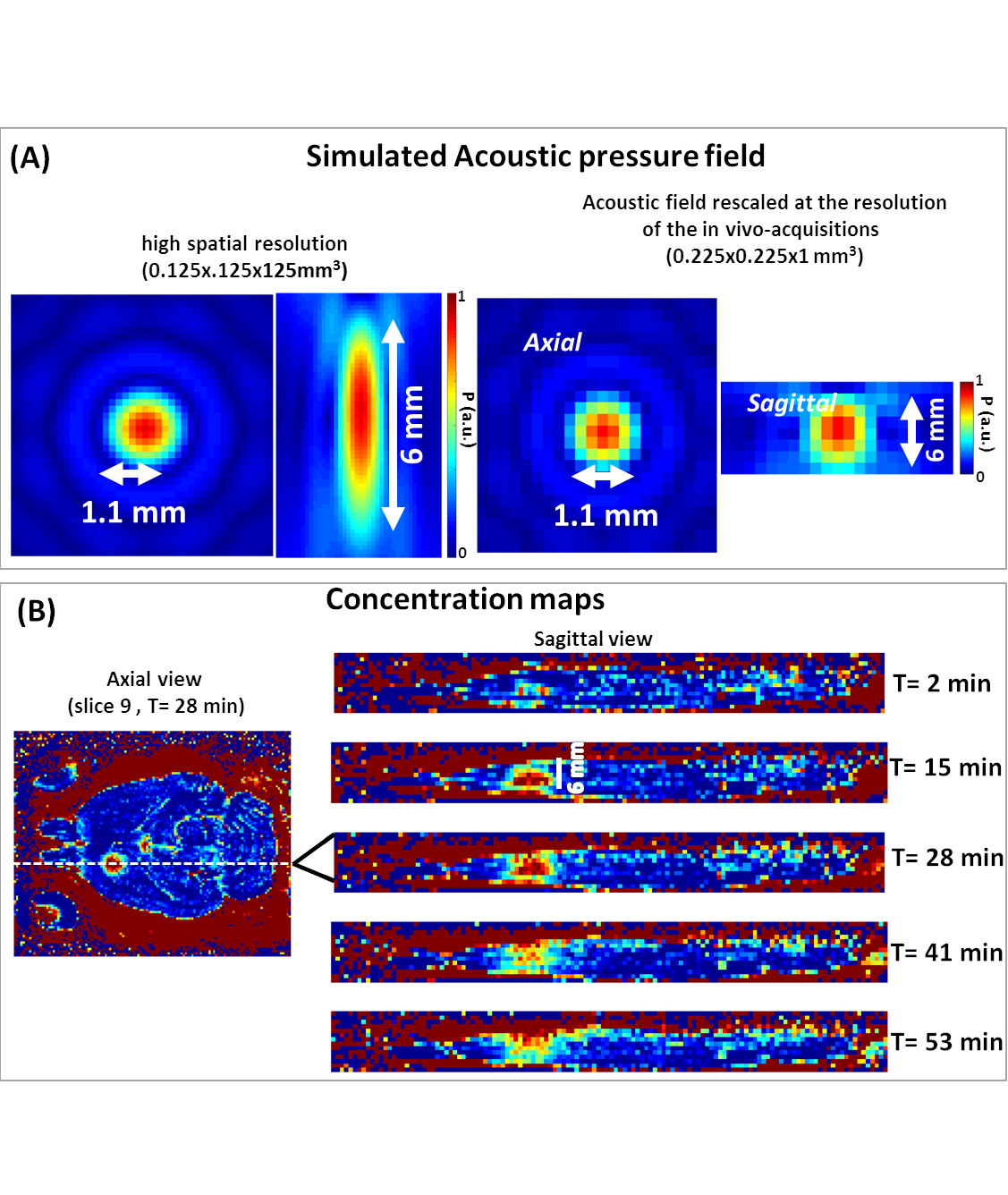


**Supplementarty Figure 1:** (A): Acoustic pressure field at 1.5 MHz. The pressure field is normalized by the maximum pressure (obtained at the focal spot). The acoustic pressure field shown on the left has been rescaled to the spatial resolution of the concentration maps. (B) Axial and sagittal views of the Gd-concentration maps: it can be noticed that the focal spot dimension along z-axis is comparable to the thickness of the rat brain in the area where the BBB was permeabilized. Moreover, the spatial resolution in this direction is much lower than the in-plane resolution (around 4.4 times). This significantly lower resolution along z-axis does not allow to precisely quantify the variations of CA concentration in this direction


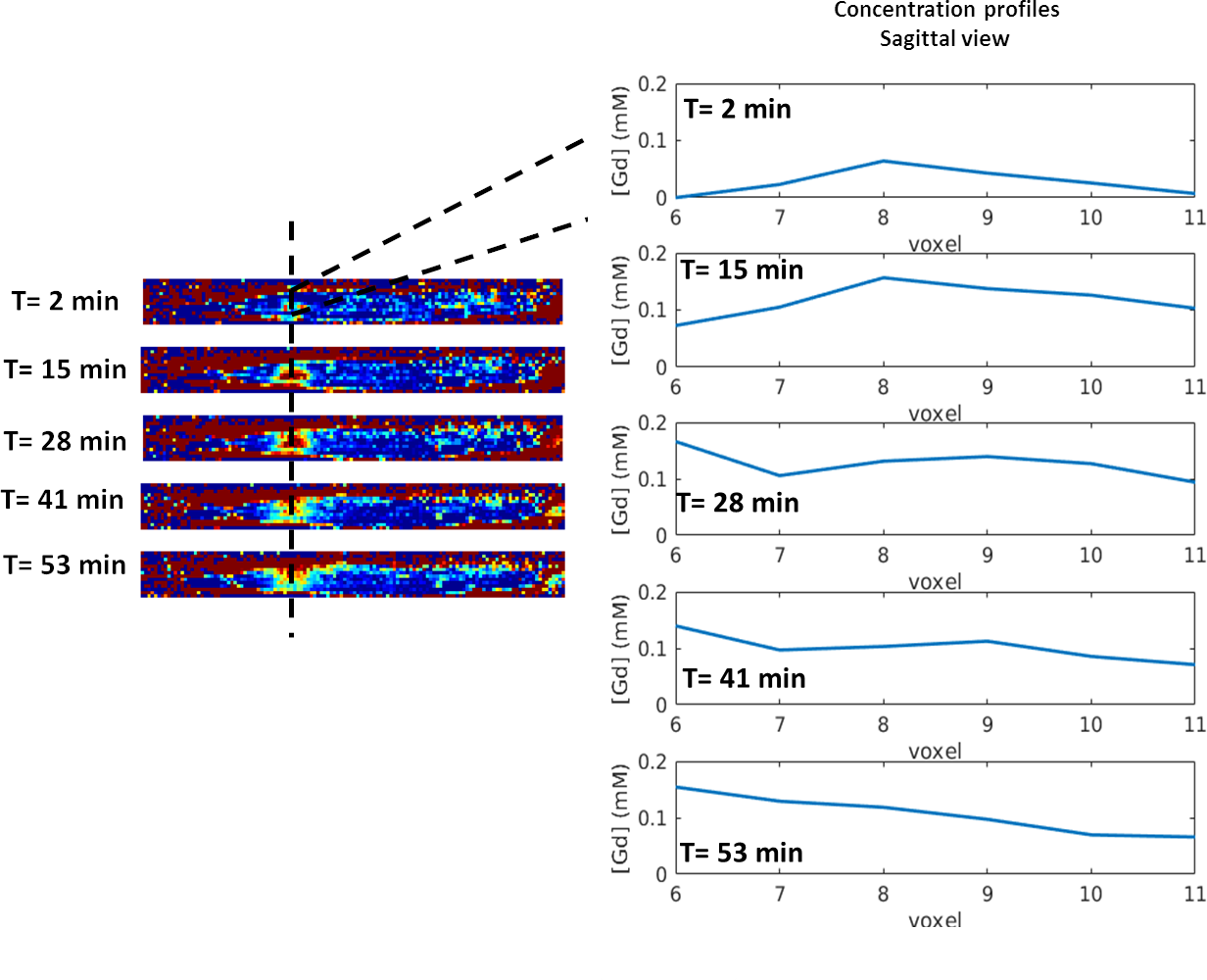


**Supplementary Figure 2:** Left: sagittal views of the Gd-concentration maps. Right: concentration profiles extrapolated from the center of the BBB opening site. These profiles show that the Gd-concentration changes only slightly with the z position. This is due to the low resolution of the Gd concentration map along z but also to the dimensions of the focal spot along this direction.


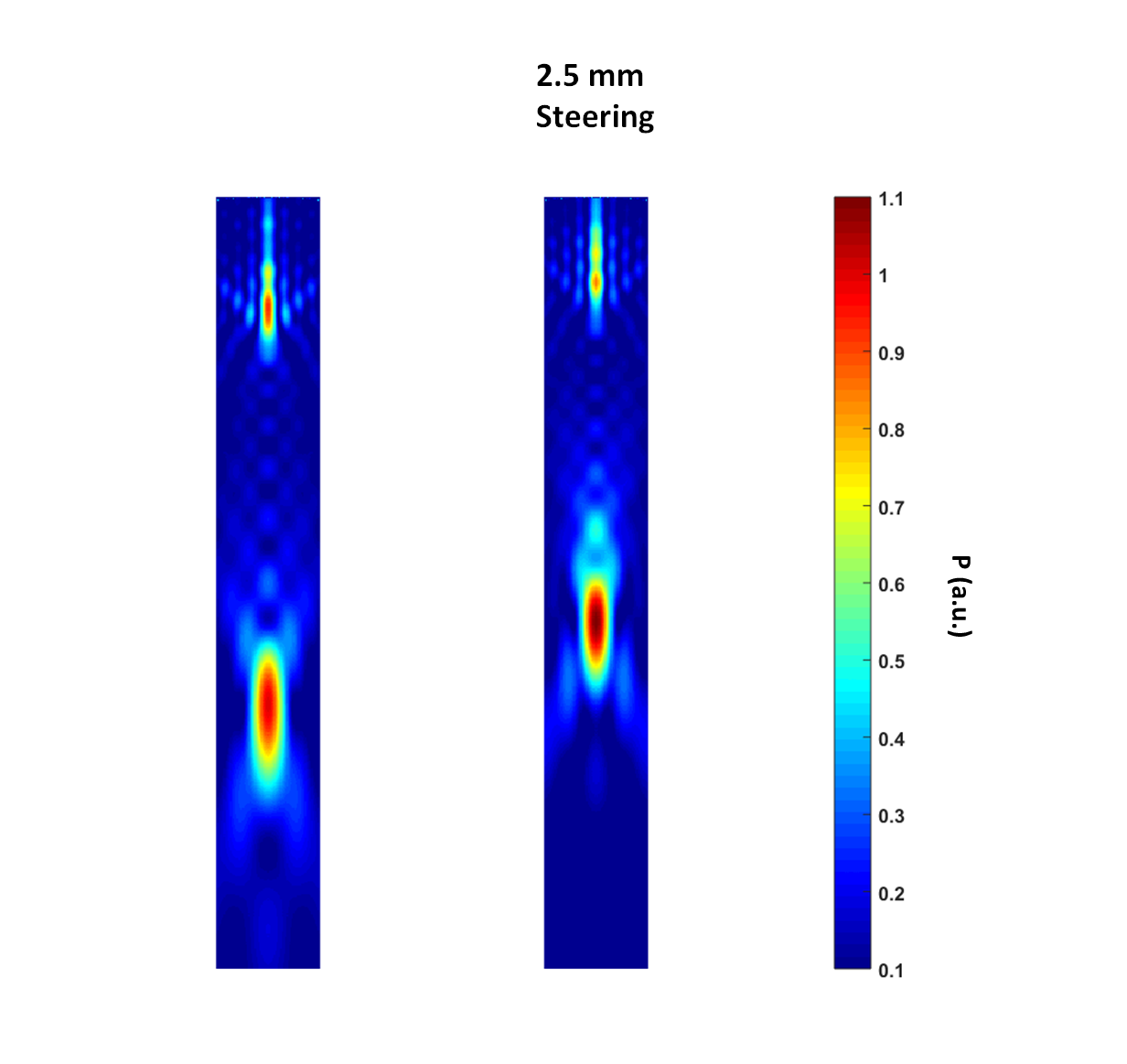


**Supplementarty Figure 3:** Acoustic pressure fields at 1.5 MHz for the concave transducer (F/D=0.8) without steering (left) and with a 2.5 mm steering toward the transducer (right). Both pressure fields are normalized by the maximum pressure obtained at the focal spot in case of the absence of steering. With a 2.5 mm steering toward the transducer, the volume of the focal spot is decreased by 20% and the maximum pressure is increased by 10% compared to the experiment without steering.


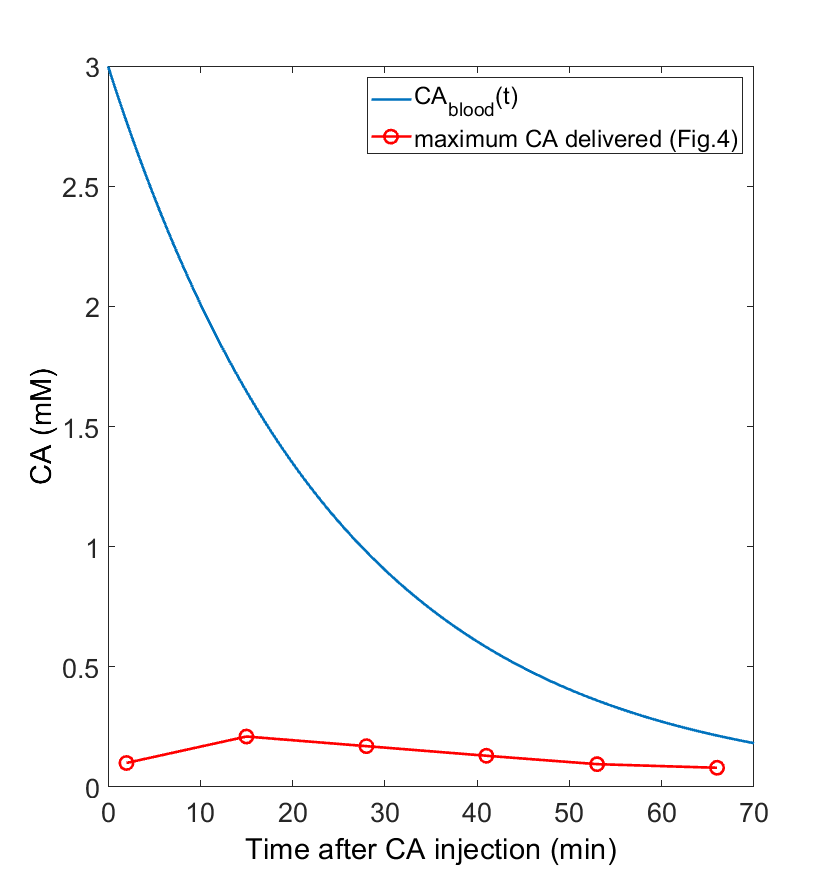


**Supplementarty Figure 4:** Figure R4: Comparison between the CA concentration in blood (picture in blue) and the maximum CA delivered during a BBB opening experiments (in red). In particular, experimental points refer to the data shown in Fig.4 while the trend of CAblood along time, *t*, has been derived through the equation CAblood(t)=CAinj∙exp(-t/b), with b=25 minutes (Aime and Caravan, JMRI, 2009).
